# Calcium intake in health maintenance – a systematic review

**DOI:** 10.3402/fnr.v57i0.21082

**Published:** 2013-05-16

**Authors:** Kirsti Uusi-Rasi, Merja U. M. Kärkkäinen, Christel J. E. Lamberg-Allardt

**Affiliations:** 1 UKK Institute for Health Promotion Research, Tampere, Finland; 2 Academy of Finland, Helsinki, Finland; 3 Department of Food and Environmental Sciences, University of Helsinki, Helsinki, Finland

**Keywords:** Calcium intake, calcium requirements, disease prevention, osteoporosis, bone mineral density, health maintenance, health outcomes

## Abstract

**Background:**

Calcium (Ca) is an essential nutrient for the human body. Despite lively research, there is uncertainty about Ca requirements in terms of desirable health outcomes including an upper intake level above which the potential for harm increases.

**Objectives:**

The aim was to conduct a review to update requirements and desirable or harmful health effects of Ca on the current scientific evidence.

**Methods:**

We searched Medline and Swemed from January 2000 to December 2011 and included all systematic reviews that reported Ca supplementation or usual Ca intake on health outcomes. Meta-analyses, randomized clinical trials and cohort studies were included in the second search between May 2009 and March 2011 and an additional search covering studies till the end of 2011. This review concentrated on studies reporting independent effects of Ca, although a few recent trials report sole effects of Ca on health outcomes, most trials use Ca in combination with vitamin D vs. placebo.

**Results:**

In total, we reviewed 38 studies addressing the effects of Ca on bone, pregnancy-related outcomes, cancers, cardiovascular outcomes, obesity, and mortality. There was a lot of heterogeneity in the study protocols, which made it difficult to draw any strong conclusions. According to the literature, high Ca intake seems to have a small positive effect on bone mineral content (BMC) or bone mineral density (BMD) in children and postmenopausal women. We did not find any consistent evidence on the effects of Ca on bone health in premenopausal women or men. Also, the evidence that Ca supplementation reduces fracture incidence is scarce and inconsistent. Maternal diet may influence the peak bone mass of offspring but more studies are required. There was no overall effect of Ca intake on cancers. Ca was associated with a decreased risk of breast cancer and a slightly increased risk of prostate cancer in two of the three studies. No associations were found with other cancers. We found no consistent association between cardiovascular outcomes and Ca intake except for blood pressure. A small decrease of 2–4 mmHg in systolic blood pressure was found in pregnant and in hypertensive subjects with Ca supplementation. Reviewed studies did not show consistent evidence relating Ca intake to either mortality or obesity.

**Conclusion:**

Based on this evidence, there is no need to change the Nordic recommendations for Ca intake. However, due to heterogeneity in the studies it is difficult to interpret the results and provide single summary statement.

This systematic literature review (SR) is a part of the NNR5 project with the aim of reviewing and updating the scientific basis of the 4th edition of the Nordic Nutrition Recommendations (NNR) issued in 2004 (Nord 2004:13) (1). The NNR5 project is mainly focused on a revision of those areas in which new scientific knowledge has emerged since the 4th edition with special relevance for the Nordic setting. A number of SRs will form the basis for establishment of dietary reference values in the 5th edition of NNR.

## Aims

The overall aim was to review the recent scientific data on requirements and health effects of calcium (Ca) to update the current dietary reference values valid in Nordic countries.

The specific objectives of the review on health effects on Ca in human nutrition are as follows:

1. Review the scientific evidence to determine, based on a set of agreed criteria, dietary reference values for Ca for different life stages (infants, children, adolescents, adults, elderly, and during pregnancy and lactation);
2. Assess the requirement of Ca for adequate growth, development, and maintenance of health; and
3. Assess the health effects of different intakes/exposures of Ca.

## Scientific background

**Fig. 1:**
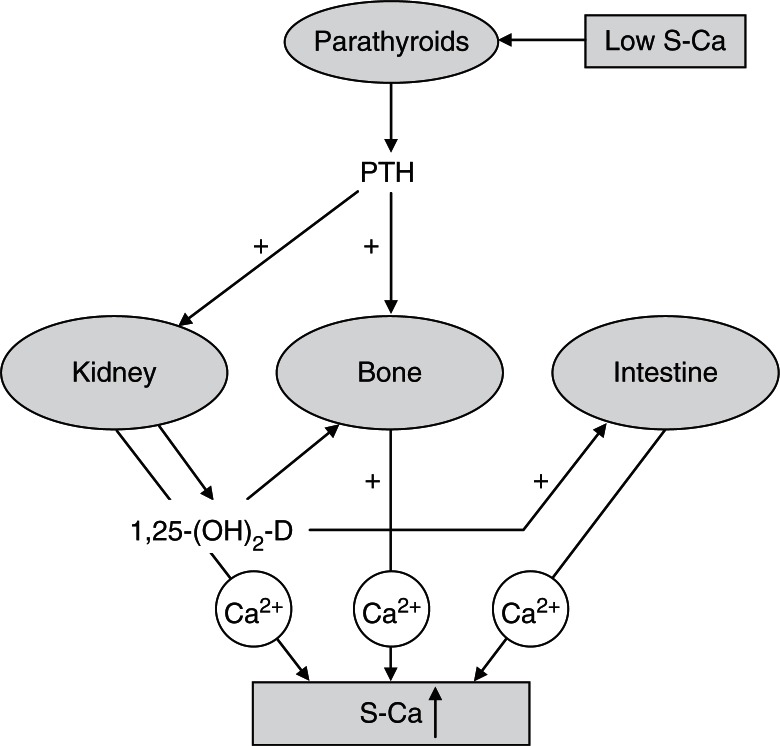
Endocrine feedback system that maintains serum calcium concentrations: involvement of 1,25(OH)_2_D and parathyroid hormone (PTH).

At full-term birth, the human infant accrues about 26–30 g of Ca, most of which is present in the skeleton as calcium hydroxyapatite (Ca_10_[PO_4_]_6_[OH]_2_), which provides the rigidity necessary for the skeleton to function mechanically. When the Ca transfer from the placenta ceases at birth, the newborn infant is dependent on dietary Ca. Ca is an important regulator of several body functions, such as muscle contraction, function of the nervous system, and blood clotting. Due to its vital importance, Ca concentration in intracellular and extracellular fluid is tightly regulated. Bone tissue serves as a reservoir and as a source of Ca for these critical metabolic needs through the process of bone remodelling. The most important regulators of Ca metabolism (Fig. 1) in humans are parathyroid hormone (PTH) and 1,25-dihydroxyvitamin D (1,25-(OH)_2_D).

Bone is a dynamic and metabolically active tissue; it is constantly renewed at an average rate of 8–10% per year, and the body's need for Ca relative to skeletal growth and remodeling varies by life stage. The major physiological activities include bone accretion during skeletal growth and maintenance of bone mass after growth is completed. Ca balance studies have shown that Ca retention is significantly higher in adolescents than in adults with the same Ca intake. Later in adult life, net Ca is lost from the body when bone formation no longer balances with bone resorption. There are two different types of bone tissue, which are formed with the same cells and matrix elements, but they differ in their structure and function. The peripheral skeleton constitutes 80% of the skeletal mass, and is composed primarily of cortical bone. About 70% of the central skeleton is, in turn, trabecular bone. Trabecular bone, with its greater surface area, is metabolically more active and is thus more responsive to changes in mineral homeostasis, whereas the cortical bone fulfills mainly the mechanical and protective functions. In adults, bone modeling occurs less frequently than bone remodeling, particularly in trabecular bone. While the turnover rate of 2–3% per year in cortical bone is consistent with maintenance of mechanical properties, the turnover rate in trabecular bone is much higher.

Dietary calcium is classically associated with dairy products, and food supplies such as milk, yoghurt, and cheese are rich sources of Ca, providing the majority of Ca in the general diet in the Scandinavian countries. In Finland (2) and Sweden (3), milk products provide more than 60% of the dietary calcium intake and in Norway (4) approximately 70%. The mean intakes in adult women in these countries vary from 800 to 1,000 mg/day and in men from 1,000 to 1,200 mg/day.

## Research/key questions for calcium

1. What is the relationship between Ca intake and different outcomes in different population and age groups?
2. What is the relationship between Ca intake and well-established markers of different functional or clinical outcomes in different population and age groups?
3. What is the effect of supplemental Ca on different outcomes in different population and age groups?
4. What is the effect of Ca intake from different sources on Ca metabolism in different population and age groups?
5. Which is the interaction of Ca intake from different sources with iron intake or iron status on health outcomes in different population and age groups?
6. Which is the UL (tolerable upper intake level) for Ca intake for different health outcomes in different population and age groups?

## Methods

### Definitions

This review focuses on publications reporting independent effects of Ca, although a few recent trials report sole Ca effects on health outcomes, most trials use Ca in combination with vitamin D vs. placebo.

The *indicators of exposure* were dietary calcium, fortified foods, and supplementation exposure.

Publication types: Questions 1–4: SRs, Cochrane database systematic reviews, meta-analyses ; Question 5: cohort studies (longitudinal or prospective studies), intervention studies; Question 6: for adverse effects: randomized controlled trial s(RCTs). Time frames for search : January 2000–April 2009; May 2009–June 2010; and July 2010–December 2011.

The following outcome measures were included:

- Pregnancy outcomes and growth
- Bone health
  - All fractures
    - Hip fractures
    - Vertebral fractures
  - BMD/osteoporosis
  - Bone mass
  - Bone quality
- Muscle strength
- All cancers
- Breast cancer
- Colorectal cancer
- Prostate cancer
- Autoimmune diseases
- Diabetes type II
- Obesity/weight control
- Total mortality
- Cardiovascular disease clinical outcomes

The search terms are defined in Appendix A.

The following *life stages* were included: infants, children, adolescents, adults, elderly, and pregnancy and lactation.

The population groups in the search were primarily Caucasian.

### Search methods and terms

Two expert reference librarians designed and conducted the electronic search strategy based on the research questions provided by the three investigators. The following electronic databases were searched: Medline and Swemed. The search was conducted using medical subject heading terms (MESH; see Appendix A). The search was done in two batches, the first covering January 2000–April 2009 and the second May 2009–June 2010 and July 2010–February 2011. A complementary search was done at the end of January 2012 covering the period between the first searches until the end of December 2011. The search is documented in Appendix A. In the first search, the investigators focused only SRs and in the second SRs and randomized control trials, published after May 2009, except the study question 5 where all types of studies were included due to the less number of hits.

Furthermore, we used snowballing for SRs and RCTs, which was not in the original search.

### Selection of articles/studies, data collection, and analyses

**Fig. 2:**
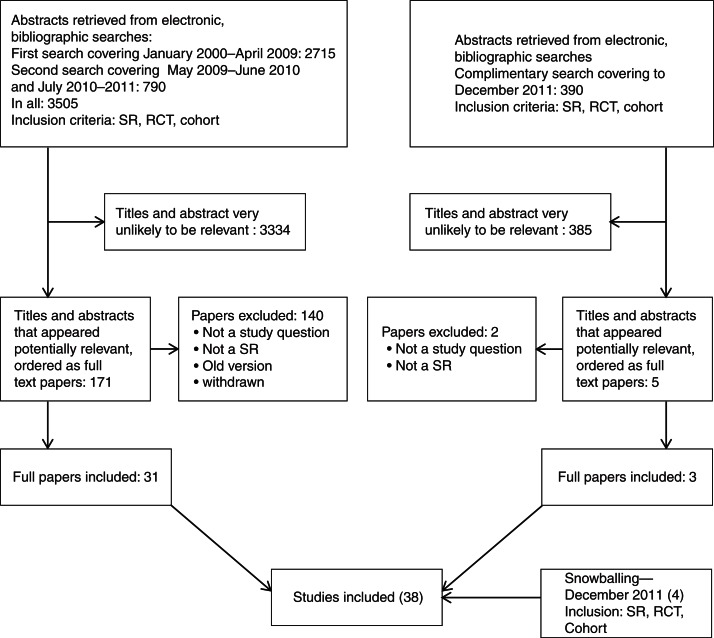
Flow chart of the study selection.

The investigators screened all abstracts individually from the searches, and later all the three investigators made a common decision on the full-text articles that had been required from the librarian. From the batches of full-text articles, we included those that met the criteria for SRs. In addition regarding study question 5, RCTs, intervention studies, and cohort studies were included. The full-text articles were examined individually and the three investigators made a common decision on which articles should be included and which to be excluded. Eligible criteria for full-text articles were SR, matching the research questions and healthy population (not patients or medication). In the case of clinical studies and cohort studies, only studies from Europe, Australia, and North America were included. We recorded the reason for rejection of all full-text articles (Fig. 2).

Three authors first independently assessed trial quality and extracted data including adverse events.

### Quality assessment of studies

The results of systematic *reviews and meta-analyses* were quality assessed and evaluated using the NNR5-modified AMSTAR quality assessment tolls and incorporated in the evidence tables. Quality assessment of the RCTs and cohort studies was done according to the NNR guidelines.

#### Participants

We focused on populations in Europe and North America. However, if other populations were included in the SRs, we were usually not able to separate them.

#### Exposure

Dietary calcium or calcium supplements such as calcium carbonate, calcium citrate, calcium chloride, or calcium phosphates.

### Study quality

There was a lot of heterogeneity in study subjects, methods, and protocols, making it difficult to interpret the results and provide single summary statement. The sources and dose of Ca varied widely among the studies, as well as the methods used to assess the amount consumed. For many risk factors, the quality and strength or direction of association varies among studies. Study cohorts were mainly Caucasian participants.

### Publication bias

Publication bias cannot be ruled out, since relevant studies were searched by the electronic databases, such as Medline and Swemed, unpublished or ongoing studies were not identified. The search was to the end of December 2011.

### Reporting and summarizing the evidence

The evidence is summarized in the evidence tables, Appendix C, tables 1–6. The included and excluded studies are listed in Appendix B.

## Effects of Ca exposure on outcome measures

### Health outcomes

Characteristics of the included studies for each health outcome are shown in Evidence Tables 1–6.

### Bone health

The results of bone health are shown in Evidence Table 1.

#### Calcium accrual and bone health in childhood

##### Calcium accrual

Since more than 99% of body Ca is present in the skeleton, an adequate Ca intake during the growth period may be critical in maximizing BMC. In their longitudinal study, Vatanparast et al. (5) reported the average accumulation of Ca (standard deviation, SD) in adolescent Caucasian boys and girls (9–18 years). Boys accrued 198.8 (74.5) g bone mass (BMC) per year, equivalent to 175.4 (65.7) mg calcium per day with the maximum accrual of 335.9 g from age 13 to 14. Girls accrued 138.2 (64.2) g BMC per year, equalling 121.8 (56.6) mg calcium per day with the maximum annual BMC accrual of 226.0 g from age 12 to 13.

##### Bone health

We found only one SR (graded A) and one meta-analysis (graded C) addressing bone health in children. We did not find any data reporting effects of Ca supplementation on bone fractures as an outcome in children.

A systematic review of Winzenberg et al. (6) included RCTs of Ca supplementation either with Ca supplements or dietary Ca compared with a placebo, with a treatment period of at least 3 months. The participants were healthy children aged 3–18 years, and bone outcomes were measured after at least 6 months of follow-up. Nineteen RCTs (*N=*2,859 participants, out of them 1,367 were randomized to supplements, 1,426 to placebo) met inclusion criteria. Ca supplementation doses ranged from 300 to 1,200 mg/day using calcium citrate, calcium carbonate, calcium phosphate, calcium lactate gluconate, calcium phosphate milk extract, or milk minerals as a source of Ca. None of these studies used dairy foods as a supplement.

There was no statistically significant effect of Ca supplementation on BMD] (mg/cm^2^) at the femoral neck (10 studies) or lumbar spine (11 studies) standardized mean differences (SMD; 95% CI) being 0.07 (from -0.05 to 0.19) and 0.08 (from -0.04 to 0.20), respectively. However, there was a small effect on total body BMC (g) (9 studies) SMD being 0.14 (0.01–0.27), as well as on upper limb density (12 studies) (0.14; 0.04–0.24). This effect is comparable to about 1.7% greater increase in supplemented groups. Only the effect in the upper limb persisted after supplementation ceased (0.14; 0.01–0.28). In the subgroup analyses, treatment effects during supplementation were greater at all sites in females than males, though not statistically significantly. Baseline Ca intake, physical activity, pubertal stage, type of supplementation (milk extract or other), duration of supplementation, and exceeding the Ca threshold did not significantly modify the effect. They concluded that although there was a small effect of Ca supplementation in the upper limb, the increase in BMD is unlikely to result in a clinically significant decrease in fracture risk.

Huncharek et al. (7) did a meta-analysis evaluating the effects of calcium/dairy supplementation on bone health using BMC (g) as the primary outcome. Initial pooling of 12 RCTs (*N=*2,460) yielded a summary mean difference (95% CI) in BMC between the Ca supplemented and placebo arms of 2.05 g (from -3.26 to 7.36 g). Owing to the heterogeneity of the pooled data, the calculated summary mean difference in BMC is of dubious validity. When the papers with low baseline Ca intake were pooled (three studies), a summary mean difference was 49.9 g (24.0–76.6 g) in total body BMC. Four RCTs contained data on lumbar spine BMC, but due to substantial differences across these reports, the authors could not further characterize the effect of Ca supplementation at the lumbar spine. They concluded that increased calcium/dairy intake significantly increases total body and lumbar spine BMC in children with low baseline intakes. However, it is improbable to demonstrate significant differences between groups when both groups are receiving physiologically adequate amounts of Ca.

In conclusion, there was a small effect of Ca supplementation in the upper limb BMD and total body BMC. From a physiological point of view Ca is essential for the skeleton. Other factors, such as vitamin D, may be crucial in the assessment of the role of Ca intake. Adequate vitamin D intake may be a crucial factor when assessing the role of Ca intake.

#### Bone health in pregnancy and fetal growth

We did not find any SR regarding Ca intake in pregnancy, but one RCT (graded C) and one longitudinal follow-up study (graded C).

In addition to genetic and lifestyle factors, adequate maternal nutrition during pregnancy may be an important contributor to fetal growth and bone health. In an RCT, healthy primiparous women (<20-week gestation) with Ca intake below 600 mg/day received either a Ca supplement of 1,500 mg/day (*N=*231) or a placebo (*N=*230) (8). There were no bone mass or density measurements of either the mother or her offspring. However, in addition to other biometric measurements, femoral and humeral diaphysis length of the fetus was measured. No differences were found in fetal biometric measurements between the supplemented or placebo groups. Similarly, neonatal characteristics and anthropometric measurements at delivery were comparable in both groups. They concluded that Ca supplementation of 1,500 mg/day in pregnant women with low Ca intake did not appear to impact on fetal somatic or skeletal growth.

A longitudinal follow-up of a Tasmanian birth cohort, mainly of Caucasian origin, was used to describe the association between maternal dietary intake in the third trimester of pregnancy and bone mass in their offspring at the age of 16. Maternal Ca intake was positively associated with lumbar spine BMD, but no association was found between femoral neck and total body bone mass. After including all significant nutrients into the same multivariate regression model, Ca intake was no more significant (9).

#### Bone health in adulthood

Optimally, after puberty and throughout most of adulthood, bone formation and resorption are balanced. During this period, bone mass is consolidated, and Ca requirements are relatively stable. Peak bone mass, the maximum amount of bone that can be accumulated, is reached in early adulthood (10, 11). The ability to attain peak bone mass is affected by genetic background and by lifestyle factors, such as physical activity and total Ca intake. Bone is a dynamic tissue, and a number of clinical studies suggest that increasing bone mass early in life has a transient effect, but does not confer protection against later bone loss and osteoporosis (12). The Ca content of bone at maturity is approximately 1,200 g in women and 1,400 g in men (13, 14). In men, this level remains relatively constant until the onset of age-related bone loss later in life, and in women until the onset of menopause. Caucasian women appear to lose as much as 3–10% of their trabecular bone per year during the first few years after menopause and about 1% of their cortical bone per year during the first decade after menopause. After this accelerated bone loss period, the loss again levels off during the postmenopausal years. Lifetime losses may reach 30–40% of peak bone mass among women and 20–30% among men (15).

#### Bone health in premenopausal women

Data are scarce with respect to bone health (mass and fractures) in premenopausal women. We did not find any SR data from this millennium reporting effects of Ca supplementation on premenopausal women's bone health.

#### Bone health in postmenopausal women

Postmenopausal women are the most common target populations for research regarding bone health. We found one SR (graded C) and two meta-analyses (graded A and C).

The SR by Waugh et al. (16) evaluated risk factors for low BMD among 40- to 60-year-old women. They found an inconsistent evidence of an association between the current Ca intake and BMD (four studies) and insufficient evidence of an association between the past Ca intake and BMD (one study). None of these studies were intervention studies, and Ca intake was self-reported. They concluded that evidence of an association between the current Ca intake and BMD was inconsistent and insufficient for past Ca intake and BMD.

The meta-analysis of Shea et al. (17) included 15 RCTs with Ca supplementations (*N*= 1,806, out of them 953 were Ca supplemented). The pooled mean (95% CI) difference in bone loss between the supplemented and placebo groups in percentage change from baseline was 1.66% (0.92–2.39%) for the lumbar spine (nine studies), 1.64% (0.70–2.57%) for the hip (eight studies), and 1.91% (0.33–3.50%) for the distal radius (six studies) favoring the Ca supplementation. They concluded that Ca supplementation alone had a small positive effect on bone density.

Chung et al. (18) included in their large review the Ottawa EPC report (19) and did an update search of RCTs published after that. The review did not separate Ca trials from Ca in combination with vitamin D trials. The Ottawa EPC report concluded that overall, there is good evidence that combined vitamin D_3_ and Ca supplementation resulted in small increases in BMD of the spine, total body, femoral neck, and total hip. The update search of Chung et al. (18) did not find evidence to change this conclusion, although quantitative synthesis was limited due to variable treatment durations and BMD sites.

#### Bone health in men

The data evaluating the effect of Ca intake on bone health in men are scarce. We found one SR (graded C) of Papaioannou et al. (20). They included 14 studies in their assessment of BMD and Ca intake in men (5 longitudinal and 9 cross-sectional studies). Cross-sectional studies showed inconsistent evidence. Five studies showed a positive association between Ca intake and BMD of the lumbar spine and hip, whereas four studies did not find an independent association between Ca intake and BMD at these bone sites. Also, the five longitudinal studies showed inconsistent evidence. In two studies, Ca intake was associated with bone loss. In particular, men in the lower quartiles of Ca intake (<1,100 mg/day) had approximately double the rate of bone loss. On the other hand, three studies did not predict bone loss.

Men with low Ca intake were underrepresented in these studies. When the participants were classified into quartiles by dietary Ca intake, the limit of the lowest quartile was <1,100 mg/day, which is mainly seen to be a sufficient Ca intake. The authors concluded that the existing data were inconsistent and did not show that Ca intake (dietary or supplements) could predict bone loss.

#### Fractures

We found one SR (graded A), three meta-analyses (one graded A, two graded C), one cohort study (graded C), and one cross-sectional study (graded C) relating Ca intake and fractures.

Shea et al. (17) found only five RCTs that measured fracture rate in a meta-analysis from 2002. The study found no significant reduction in either vertebral fracture risk ratio (RR, 95% CI) being 0.77 (0.54–1.09) or in non-vertebral fractures, RR 0.86 (0.43–1.72). They also concluded that the magnitude of reduction in fracture risk with Ca supplementation alone remains an open question.

Tang et al. (21) reported in their meta-analysis whether Ca or Ca in combination with vitamin D prevents fractures and bone loss. Twenty-nine studies were included in the analyses, out of them 17 reported all types of fractures as an outcome and 24 reported BMD. In total, 64,897 individuals 50 years or older, of them 92% women, were included. In 16 trials, participants received Ca-only supplements. Effect of Ca only on fracture risk reduction (RR; 95% CI) was 0.90 (0.80–1.00). The difference in RR between Ca-only supplementation and Ca with vitamin D combination was very small (0.90 vs. 0.87) and was not significant. The treatment effect was greater in participants whose daily intake was low (less than 700 mg/day). They concluded that the fracture risk reduction was greater in the trial in which the compliance rate was high. In addition, the treatment effect was better with Ca doses of 1,200 mg/day or more.

Instead of all types of fractures, Bischoff-Ferrari et al. (22) assessed in their meta-analysis the relationship of Ca intake to the risk of non-vertebral and hip fractures. In the included five RCTs, Ca supplementation varied between 800 and 1,600 mg/day and was compared with the placebo. Among 6,740 participants (5,666 primarily postmenopausal women and 1,074 men), the pooled risk ratio (RR; 95% CI) for non-vertebral fractures was non-significant, 0.92 (0.81–1.05). The pooled risk of four RCTs with separate results for hip fracture (*n=*6,504, 139 hip fractures) was 1.64 (1.02–2.64). Pooled results from seven prospective cohort studies (*N=*170,991 women, 2,954 hip fractures) found no association between Ca intake and hip fracture risk (RR per 300 mg total Ca per day=1.01; 0.97–1.05). They concluded that Ca intake was not significantly associated with hip fracture risk in women or men based on pooled results from prospective cohort studies. The pooled results from RCTs showed no reduction in hip fracture risk with Ca supplementation, and an increased risk was possible.

The Ottawa EPC report (19) was included in the SR of Chung et al. (18). The authors concluded that Ca supplementation in combination with vitamin D (most studies used D_3_) is effective in reducing fractures and falls in institutionalized populations, but there is inconsistent evidence in reducing falls in postmenopausal women and older men. The update search of Chung et al. (18) identified one new RCT of female Navy recruit, aged 17–35, which showed that vitamin D (800 IU/day) in combination with Ca (2,000 mg/day) supplementation can reduce the risk of stress fractures from military training compared to placebo.

In a Swedish cohort study, Warensjö et al. (23) evaluated the association between Ca intake and risk of fractures (*n*=61,433 women born between 1914 and 1948) and osteoporosis (subcohort of 5,022 women). The participants were divided into quintiles by Ca intake, and the third quintile was used as a reference group (range of mean Ca intake: 882–992 mg/day). The HR for the first event of any fracture was 1.18 (1.12–1.25) and for hip fracture 1.29 (1.17–1.43) in the lowest quintile (Ca intake<751 mg/day). The risk for osteoporosis was also higher the OR being 1.47 (1.09–2.00). The highest quintile of Ca intake did not further reduce the risk of fractures of any type, or of osteoporosis, but was associated with a higher rate of first-event hip fracture (HR 1.19; 1.06–1.32).

In their cross-sectional study, Zhong et al. (24) evaluated the association of Ca intake and dietary protein intake with risk of fractures in postmenopausal women aged 50 years or older. They did not find a relationship between total Ca intake and risk of fractures. However, the cross-sectional nature does not permit assessment of causality, and establishing the effect of Ca supplementation on fractures would require large, relatively long trials measuring fracture incidence.

### Pregnancy-related outcomes

We found two high-quality and one good quality systematic reviews on the effects of Ca during pregnancy (Evidence Table 2). The other included studies, one RCT and one cohort study, had serious flaws in their study design. Only the results of at least good quality studies are reviewed here.

#### Offspring

Buppasiri et al. (25) reviewed 21 RCTs involving 16,602 pregnant women. They found a significant increase of approximately 65 g (95% CI 16–114 g) in birth weight in children whose mothers had used Ca supplements during pregnancy compared to babies of non-users (19 trials, 8,287 women; Evidence Table 2). No significant differences were, however, found in infant with low birth weight between the two groups.

In the systematic review by Bergel et al. (26) of two RCTs and three observational studies, no consistent effect was found on the blood pressure of infants. However, in children aged 1–9 years, higher maternal Ca intake (dietary or from supplements) was associated with lower systolic blood pressure (mean: 1.92 mmHg, 95% CI 0.71–3.14 mmHg). No association with diastolic blood pressure was found. This finding was supported by another systematic review of Hofmeyr et al. (27), which found that the risk of childhood systolic blood pressure greater than 95th percentile was reduced (514 children: RR 0.59, 95% CI 0.39–0.91).

#### Mother

Hofmeyr et al. (27) reviewed 13 randomized trials comparing at least 1 g daily of Ca during pregnancy with a placebo. They found that the risk of high blood pressure was reduced by 35% (12 trials with 15,730 women, RR=0.65, 95% CI 0.53–0.81), the effect being greatest in women with low baseline Ca intake. In four small trials among women at high risk of developing pre-eclampsia (568 women: RR 0.45, 95% CI 0.24–0.83), the risk of pre-eclampsia was reduced by 55%.

The results on preterm birth were inconsistent. Hofmeyr et al. (27) reported that the average risk of preterm birth was reduced in the Ca group overall (11 trials, 15,275 women: RR 0.76; 95% CI 0.60–0.97). In a systematic review by Buppasiri et al. (25), there were no statistically significant differences between women who received Ca supplementation and those who did not in terms of reducing preterm births (less than 37-week gestation) (RR 0.90; 95% CI 0.73–1.11; 12 studies, 15,615 women; random-effects model) and also in less than 34-week gestation (RR 1.11; 95% CI 0.84–1.46; three trials, 5,145 women).

#### Lactation

We did not find any SR regarding relationships between Ca intake and lactation.

### Cancer

Nine studies (five SR, one meta-analysis, three cohort studies) with cancer as an outcome were included (Evidence Table 3). Of these 9 studies, 2 SRs were ranked as high quality and three cohorts as good quality studies. Ca intake did not have a consistent association with different types of cancers; some of the studies showed protective effect and in some studies Ca was associated with an increased risk of some type of cancers.

In the three studies of which one was meta-analysis (28), one systematic review (18) and one cohort study (29) having *breast cancer* as an outcome, Ca intakes were associated with a lower risk of breast cancer (28) in premenopausal women only (18) and tended to have an association in the third study (29).

Ca intake was associated with lower risk of *recurrence of colorectal adenomas* in one high-quality SR (30) and in another SR (31) but not with the risk of *colorectal cancer* in SR (30) in the general population.

High Ca intake was associated with slightly increased risk of *prostate cancer* in two of the three studies (18, 32, 33). Only one of these studies was ranked high quality.

No significant associations were found with *endometrial cancer* (34), *lung cancer* (35), or *total cancer* (18).

### Cardiovascular outcomes

Thirteen studies (seven SRs, three RCTs, three cohort studies) addressed different types or cardiovascular outcomes (Evidence Table 4). Three of the SRs were ranked giving high-quality evidence; one SR, one RCT, and one cohort were ranked as good quality studies. No consistent evidence of Ca intake with other cardiovascular outcomes other than systolic blood pressure was found in these studies.

Ca intake or Ca supplementation was not significantly associated with increased or reduced risk of *aortic valve calcification*, *coronary artery calcification* (CAC) (36), increased *serum lipids* (36, 37), *stroke or death* (36, 38) or *cardiovascular disease mortality* (39), *atherosclerosis vascular disease* (40), *cardiovascular events* (41) or *abdominal aortic calcification or coronary aortic calcification* (42). In one study (43), Ca supplementation was associated with *increased risk of myocardial infarction* but not in the others (40, 41). In the only included study that had diabetes as an outcome (44), the higher Ca intake was associated with reduced risk of type 2 diabetes.

The cardiovascular outcomes with Ca intake during pregnancy are reported in pregnancy-related outcomes.

Ca supplementation lowered *systolic blood pressure* with 2–4 mmHg in hypertensive and in pregnant women (18, 27, 45) in three high-quality SRs. One RCT found a small effect on blood pressure in those with below median Ca intakes (37).

### Obesity and control of body weight

Three systematic reviews, of these two high quality (18, 46, 47) and two randomized clinical trials (37, 48) with body weight and/or obesity as an outcome, were included in this review (Evidence Table 5). No consistent effect was found. In only one SR (seven RCTs, 794 subjects) (47), a mean difference of −0.74 and −0.93 kg body fat favoring higher Ca intake was found. The studies reviewed did not show convincing evidence or a favorable effect of Ca intake on body weight.

### Total mortality

Mortality as an outcome was assessed in five studies (two SRs and three cohort studies), of which one SR was ranked high quality (Evidence Table 6). No consistent effect was found of Ca intake on death of various causes. The results varied from increased risk of *death of cardiovascular causes* (43), to no effect (18, 43), to about 10–25% decreased risk *all-cause death* (38, 39, 49) with higher Ca intake.

#### Upper intake level

We did not find any dose–response data to evaluate the safety limits of upper intake level. We included only clinical outcomes, such as all-cause mortality, cancer, and cardiovascular events. The results of these outcomes have been described in previous sections. Briefly, we did not find that calcium/dairy intake is associated with an increased risk of mortality. On the contrary, all-cause mortality was lowest in those with the highest use of Ca (38, 39, 49). The results with relationship to cancer showed either no relationship or high Ca intake having a protective association. Prostate cancer was an exception with inconsistent results. However, observational studies suggested an increased risk of prostate cancer among men with high daily Ca intake of more than 2,000 mg (32, 33).

## Discussion

The aim of this review is to find a scientific base for a Nordic recommendation for dietary intake of Ca. We analyzed the literature on the relationships between Ca intake and different health outcomes. We focused on published systematic reviews but included a few RCTs and cohort studies. Some of the SRs included observational studies as well as RCTs. The quality of the SRs varied from A to C, while none of the other type of studies reached the score of A.

### Main findings in relationship to the research questions

#### Dose–response assessment including exposure range

The difference in Ca intake between the studies, some estimating total Ca intake, some concentrating on supplemented Ca, and some neglecting the supplemental use assessing only dietary intake, complicates the interpretation of Ca effects. Also, the methodology of assessing dietary Ca intake varies widely from dietary records of several days and validated food frequency questionnaires (FFQs) to enquire the use of milk in general. Amount of milk and milk products, the richest and most important Ca source in Nordic countries, is fairly stable simplifying the estimation of Ca intake, which is still challenging. Although the participants are willing to record their diet to the best of their ability, there may be a discrepancy between actual and reported Ca intake. The most common method is an FFQ with varying number of items on the list. If the FFQ is validated for the study at issue, it mainly works well, but often the FFQ is done for different purposes, for different age groups or even for different ethnic groups. Single 24-h recalls have a problem, because individual estimates may not represent usual intake.

The use of terminology is not always clear. Some studies use the term Ca supplementation but also include the use of vitamin D in combination with Ca supplements. Thus, it is not possible to evaluate the sole Ca effect. Owing to increasing interest in vitamin D and health, recent studies focus on the use of Ca in combination with vitamin D, or the comparison is done between Ca and CaD without pure placebo groups. Self-reported uses of Ca supplements may have been asked with one question without any further enquiry about name or quantity of the ingredient.

### Health outcomes

#### Bone health

Methodological inadequacies are the biggest limitation in evaluating factors affecting bone health. Dual energy X-ray absorptiometry (DXA) provides a reasonable overall description of bone density, which is a measure of mineral content of the bone, but overlooks geometric alterations that can influence bone strength. However, it is ultimately the whole bone structure, that is, its shape and size, mean cortical thickness, cortical, and trabecular density not its mass, mineral content, or areal density (BMC or BMD) that determines bone mechanical competence. Although BMD correlates directly with the change in bone mass, it does not take into account possible dimensional, material, or structural properties of bone, and is subject to inherent uncertainty (50, 51). A consideration of geometric properties emphasizes that both the amount and location of bone mineral are important. Age-related loss of bone mass is not necessarily accompanied by a proportionate age-related loss of bone strength because geometric adaptation may compensate for the loss. Age-related cortical thinning of long bone shafts, for example, is associated with increased bone girth, a compensatory change that tends to increase structural rigidity (52).

Although BMD measured with planar DXA is the most prevalent outcome in bone research, it does not represent well all determinants of bone geometric properties. However, thus far there is limited research evaluating effects of dietary or supplemental Ca on bone strength or structure.

In their reviews among children, Winzenberg et al. (6) included studies with Ca supplements, while Huncharek et al. (7) reviewed works with dietary Ca and dairy products. Ca intake affected bone mass positively, and although the effect was small, Ca intake was crucial in childhood and youth. Furthermore, the benefit was especially seen in children with low baseline Ca intake. It is likely that Ca intake is a necessary but insufficient condition for the development of a strong skeleton. However, the results do not support the general use of Ca supplementation in healthy children. Adequate vitamin D intake may have an essential factor when assessing the role of Ca intake. There are also some findings that low Ca intake is more harmful when associated with low vitamin D status (53).

Most studies on the effects of Ca intake on adult bone mass concerned postmenopausal women. We were not able to find any recent SRs about Ca intake and bone health in premenopausal women after the review of Welten et al. from the year 1995, where they analyzed the relationship between Ca intake and bone mass among young adults between 18 and 50 years of age (54). They included 33 studies and only 4 of them were intervention studies. The cross-sectional studies in women suggested a positive association between Ca intake and bone mass. Also, the few intervention studies found a consistent positive effect of a Ca supplement of about 1,000 mg/day on bone mass. These studies suggest that Ca intake should be at least 800 mg/day to optimize bone mass.

Due to high heterogeneity, inconsistent results, lack of RCTs, and limited number of other kind of studies (one SR included five longitudinal and nine cross-sectional studies), it is not possible to make any conclusions in the case of men.

In postmenopausal women, a positive treatment effect on BMD was evident in most studies. Ca supplementation had a relatively small, but possibly meaningful, effect in reducing bone loss (17). Ca in combination with vitamin D_3_ supplementation resulted in small increases in BMD of the spine, femoral neck, and total hip. Based on included trials, it was less certain whether vitamin D3 supplementation alone has a significant effect on BMD (18, 21).

#### Fractures

The evidence that Ca supplementation reduces fracture incidence is less and inconsistent. Pooled results from cohort studies did not find significant associations between the total Ca intake and hip fracture risk in women or men (22). Also, a longitudinal follow-up of a Swedish cohort showed increased risk of fragility fractures and osteoporosis in women with Ca intake below 751 mg/day. In that cohort, women with the highest Ca intake (>1,137 mg/day) had increased risk of first-event hip fracture (23). On the other hand, in the meta-analysis of Tang et al. (21), the treatment effect was better with doses at or above 1,200 mg/day than with lower doses, and in individuals who were elderly, had low dietary Ca intake (<700 mg/day), or were compliant with Ca supplementation (>80%) (21). However, participants were mainly women, and the data are limited for men. Furthermore, Ca supplementation with vitamin D may be effective in reducing fractures in institutionalized populations although the influence remains controversial in general population (18).

As a general conclusion, Ca is essential for bone health. Although Ca is only one factor contributing to bone mass and strength, it is essential for correct bone development, and especially in individuals with a low intake, sufficient Ca intake will reduce, but not prevent bone loss. Increased Ca intake suppresses the number of bone-remodeling sites and augments premature mineralization of immature bone, leading to an apparent increase in bone density. Net bone accumulation will be greater as Ca intake increases to the point of genetic program governing growth. Further increases in Ca intake will produce no further bone accumulation. This may be different for different stages of growth (55). It has also been found in postmenopausal women that the rate of bone loss is significantly lower in the first year of Ca supplementation than in the second year, especially at regions where more than half of the bone content is of the trabecular type. Furthermore, the long-term (3–4 years) preservation of bone by Ca supplements has been found mainly at sites with a predominance of cortical bone, such as the radius and total body, with an annual turnover rate of only 2–3%. This slow rate of cortical bone turnover implies that a steady state is reached many years later than in trabecular bone. The long-term effect of additional Ca on bone density and fracture prevention is therefore not readily revealed by or extrapolated from randomized bone density studies (56).

Ca supplementation may transiently increase BMD by reducing the rate of bone remodeling. Increases in bone mass appear to occur primarily in cortical bone sites, are most apparent among populations with low Ca intake, and do not seem to persist beyond the Ca supplementation period.

#### Pregnancy-related outcomes

##### Offspring

The evidence regarding maternal Ca intake and offspring's health is scarce and contradictory. In their RCT, Abalos et al. (8) did not find any difference in fetal growth between fetuses of women with or without Ca supplements. However, a small increase of 65 g in birth weight was found in SR of Buppasiri et al. (25). This increase is most likely not clinically important, and due to high heterogeneity this result must be interpreted with caution. There was no difference in other pregnancy and infant outcomes (25).

Some results support an association between maternal Ca intake during pregnancy and offspring systolic blood pressure among children (26, 27). The evidence is more consistent among children older than 1 year (26).

##### Mother

Ca supplementation is associated with a significant protective benefit in the prevention of pre-eclampsia especially in women with low baseline Ca intake (18, 27). The average risk of high blood pressure was also reduced with Ca supplementation compared with a placebo (18, 27). Since pre-eclampsia is a dangerous life-threatening disorder, in which hypertension arises, Ca supplementation may be one factor in reducing the risk of this serious medical condition. Since pre-eclampsia is a dangerous life-threatening disorder, in which hypertension arises, in women with low Ca intake Ca supplementation may be one factor in reducing the risk of this serious medical condition.

However, there was no evidence that Ca supplementation had any effect on maternal weight gain during pregnancy (25).

##### Lactation

Profound changes in Ca metabolism and bone mineral status accompany pregnancy both during gestation and after delivery and lactation. Accumulating data suggest that the losses are independent of maternal Ca intake, and largely reversible in subsequent lactation and after the end of breast-feeding, possibly in connection with the return of menses (57, 58). This does not imply that good nutrition, including the maintenance of adequate Ca intake, is important during lactation. However, the accumulating data suggest that breast-feeding women need not consume excess Ca (59).

##### Cancer

In general, no association has been found between increasing Ca intakes and cancer incidence or mortality (Chung et al. 2009). However, there are inconsistent findings considering different cancer types. In women, there was a trend toward lower breast cancer risk for those with high Ca intake (18, 29, 60).

The results concerning prostate cancer are inconsistent showing slightly increased association, no association or inverse association between Ca intake and risk of prostate cancer (18, 32, 33). The results of Kristal et al. do not simplify the interpretation. They report that dietary Ca intake was positively associated with low-grade cancer but inversely associated with high-grade cancer.

The results are not unambiguous in aspects of colorectal cancer, either. Although there are some findings that high Ca intake may contribute, to a moderate degree, to the prevention of adenoma polyps recurrence especially in patients with a history of adenomas, no apparent effect was seen in advanced adenomas or in populations at lower risk (18, 30, 31). More evidence is needed to recommend the general use of Ca supplements to prevent colorectal cancer.

The current evidence for a role of Ca in endometrial carcinomas is too limited to draw any conclusions (34). Similarly, more evidence is needed about the relationship between Ca intake and lung cancer, although increased total Ca intake reduced lung cancer risk in a subgroup of current smokers and a beneficial trend was seen in women (35).

#### Cardiovascular outcomes

##### Hypertension and blood pressure

Fairly consistent evidence suggests that Ca supplementation lowered systolic blood pressure among hypertensive adults, but no significant effect has been found on diastolic blood pressure or in normotensive individuals (18, 37, 45).

More data are needed about influences of age, sex, Ca dose, background dietary Ca, and supplement versus dietary source on blood pressure.

No consistent evidence of the relationship between Ca intake and other cardiovascular outcomes was found (36, 37, 39–41, 43). Even a possible increased risk concerning cardiovascular events is inconclusive.

##### Cardiovascular events

Recently, concern has arisen of an increased risk of cardiovascular events associated with Ca supplementation. Bolland et al. (43) suggested an upward trend in cardiovascular events in older people taking Ca. However, there are several limitations. Cardiovascular events were not a primary outcome, the events may not have been well adjudicated, the studies included are small and the event frequency is low. Moreover, the total Ca intake was not reported, and the supplementation was generally 1,000–1,200 mg/day. The events may therefore be associated with intakes higher than the supplemented dose, perhaps more than 2,000 mg of calcium per day. So, it is difficult to conclude that Ca intakes per se in the range of 1,000–1,200 mg/day can be associated with cardiovascular events (60). There are also opposing findings with no increase in cardiovascular events (40, 41), and supplements may even improve some vascular risk factors, e.g., blood pressure (37, 45).

#### Diabetes

Although Pittas et al. (44) found an inverse association between Ca or dairy intake and type 2 diabetes or metabolic syndrome, they concluded that the evidence is limited because most observational studies are of low quality, whereas duration of intervention studies was short, included few participants, or did post-hoc analyses.

#### Body weight/obesity

No convincing findings regarding benefits of Ca supplementation on body weight loss or weight maintenance were found. Ca supplementation did not alter weight or fat gain (18, 37, 46, 47).

#### Total mortality

The evidence relating total mortality is inconsistent. There is some proof about increased death of cardiovascular events (43), also a protective effect of Ca intake, especially on all-cause mortality (39, 49, 61), or no effect (18, 43). There are considerable differences between the works showing a protective influence, which complicates the interpretation. Mursu et al. (49) reported the use of Ca supplements in relationship to total mortality in older women, whereas Kaluza et al. (39) assessed dietary Ca intake in middle-aged or older men. The follow-up time of 65 years of van der Pols et al. (38) is considerable. Although the results suggest a beneficial effect of high dairy consumption in childhood on total mortality in adulthood, environmental and socio-economic circumstances have changed during the follow-up period, but these changes are impossible to be considered in the analysis. A much smaller challenge is the difficulty in separating the Ca effect from the total diet. One possible interpretation is that high dairy consumption is one factor in a healthy balanced diet. Also, it is difficult to compare the results of Bolland et al. (43) (supplement users, mainly women) and Chung et al. (18), whose result is based on total Ca intake in one cohort of adult men and their spouses.

#### Adverse effects

Ca is mainly thought of to be a safe nutrient to be used as a supplement, although recently there has been much of debate in the literature about the possible adverse effects of high Ca supplementation. Discordant data provided limited proof of the unfavorable effects of high calcium/dairy intake. So far, only a few studies on the whole have reported adverse intestinal discomfort, such as constipation and gas, being apparently related to the Ca supplements (18).

Kidney stone formation is an adverse outcome, most notably among postmenopausal women. However, the levels of Ca intake that may cause kidney stones within a normal population cannot be specified with certainty and are known to be vary depending on a number of factors, including baseline renal function, pre-existing disease conditions, and interactions with drugs (61).

In the past, milk-alkali syndrome was often a side effect of treating peptic ulcer disease with antacids containing Ca. It is rarely seen today, because newer, better medications that do not contain Ca are available for treating ulcers. A more common scenario today is when someone takes excess calcium carbonate in an attempt to prevent osteoporosis. Calcium deposits in the kidneys and in other tissues can occur in milk-alkali syndrome. High levels of vitamin D can worsen this condition. This has been reported in persons who take 2 g or more of calcium per day. Though the modern management of peptic ulcer disease has radically changed over the past decades, milk-alkali syndrome may still occur, especially in patients who self-medicate for symptoms of dyspepsia (62).

#### Doses

The total Ca intake has not been assessed in most of the studies. There is a huge variation in daily Ca intake. Few studies have assessed total Ca intake including dietary and supplemented Ca. Many studies using supplements have not asked about dietary Ca intake, but on the other hand some studies assessing dietary Ca intake have neglected the possible use of supplements. Dose–response studies have not been performed.

#### Study duration

Study duration varied from a short few weeks’ interventions to follow-ups of several years. Short interventions may be enough to show changes in blood pressure or serum lipids, whereas evaluation of Ca intake on bone density takes at least a few months and cardiovascular events, cancer or fragility fractures need an exposure of several years.

#### Heterogeneity

Owing to heterogeneity in the studies, it is difficult to interpret the results and provide single summary statement. The sources and dose of Ca vary widely among the studies, as well as the methods used to assess the amount consumed. For many risk factors, the quality and strength or direction of association varies among studies. Study cohorts were mainly Caucasian participants.

Substantial differences exist across the studies in many important factors. Many of the studies enrolled participants with sufficient or near-sufficient Ca intake, whereas in others baseline Ca intake was well below the recommended level, especially in reports from Asian populations which were included in the SRs. Also, the relatively large range in supplemental doses may complicate the comparison. Compliance has mostly not been reported or taken into account.

#### Limitations

Most studies tested Ca supplementation, not total Ca intake, i.e., dietary Ca and supplementation. However, the effect of Ca intake may differ according to baseline Ca intake. Evaluation of Ca intake in groups, where mean Ca intake is above the recommended daily intake may mask individuals with low habitual intake or Ca deficiency. In addition, several studies examined Ca with vitamin D supplementation, or the participants are allowed to use multivitamins and even other Ca or vitamin D supplements were used simultaneously, which makes it difficult to interpret the results.

## Conclusions

We did not find solid evidence for changing the Nordic recommendations from 2005. Although calcium/dairy supplementation had a marginal impact on bone health in the studies, milk and dairy products has to be considered to be an optimal source of Ca in the Nordic setting with important effects on bone health. Regarding children, the effect of Ca supplementation on the skeleton remained small, but most studies included children with adequate Ca intake. It is likely that Ca supplementation has a much greater impact on bone mass accretion in children with low baseline Ca intake. Similarly in adults, the treatment effect seems to be greater in participants with low daily Ca intake.

It is also difficult to separate the effect of Ca from the combination of Ca and vitamin D.

In addition to skeletal health, there is evidence that high Ca intake has a protective influence in reducing blood pressure and pre-eclampsia. The results suggesting that high supplementary Ca intake has a harmful effect on health outcomes, especially on cardiovascular health or prostate cancer are not solid. However, Ca supplementation does not seem to affect weight loss or weight maintenance, and regarding relationships between Ca intake and other cancers, the evidence is inconsistent or contradictory.

However, if individual dietary Ca intake exceeds the recommendation (depending on the age group), there is no need to recommend Ca supplements for safety reasons. We recognize that these conclusions, which are based on the current evidence, might change as additional data about long-term effects of Ca supplementation become available.

## Implications for research

1. The adverse effects of increased Ca intake on health outcomes.
2. Studies including both dietary and supplemental Ca.
